# Pathological mechanisms and future therapeutic directions of thrombin in intracerebral hemorrhage: a systematic review

**DOI:** 10.3389/fphar.2024.1293428

**Published:** 2024-04-18

**Authors:** Chenxi Tao, Yuanyuan Li, Na An, Haoqi Liu, Zhenhong Liu, Yikun Sun, Ying Qian, Na Li, Yanwei Xing, Yonghong Gao

**Affiliations:** ^1^ Key Laboratory of Chinese Internal Medicine of Ministry of Education, Dongzhimen Hospital, Beijing University of Chinese Medicine, Beijing, China; ^2^ Institute for Brain Disorders, Beijing University of Chinese Medicine, Beijing, China; ^3^ Guang’an Men Hospital, China Academy of Chinese Medical Sciences, Beijing, China

**Keywords:** intracerebral hemorrhage, thrombin, secondary brain injury, inflammation, blood-brain barrier, neuronal damage, systematic review

## Abstract

Intracerebral hemorrhage (ICH), a common subtype of hemorrhagic stroke, often causes severe disability or death. ICH induces adverse events that might lead to secondary brain injury (SBI), and there is currently a lack of specific effective treatment strategies. To provide a new direction for SBI treatment post-ICH, the systematic review discussed how thrombin impacts secondary injury after ICH through several potentially deleterious or protective mechanisms. We included 39 studies and evaluated them using SYRCLE’s ROB tool. Subsequently, we explored the potential molecular mechanisms of thrombin-mediated effects on SBI post-ICH in terms of inflammation, iron deposition, autophagy, and angiogenesis. Furthermore, we described the effects of thrombin in endothelial cells, astrocytes, pericytes, microglia, and neurons, as well as the harmful and beneficial effects of high and low thrombin concentrations on ICH. Finally, we concluded the current research status of thrombin therapy for ICH, which will provide a basis for the future clinical application of thrombin in the treatment of ICH.

## 1 Introduction

ICH, the most prevalent subtype of hemorrhagic stroke, is a critical illness that causes a substantial burden of severe disability or death (An et al., 2017). The case fatality rate of ICH is high (59% at 1 year and 70% at 5 years), with only 12%–39% of survivors achieving long-term functional recovery and independence (An et al., 2017; Wilkinson et al., 2018). Consequently, more than 80% of ICH survivors suffer from permanent disabilities (Ren et al., 2020). At present, acute ICH can be managed through interventions aimed at preventing hematoma expansion, controlling intracranial pressure, and treating edema. Despite these measures, clinical outcomes frequently fall short of optimal, highlighting persistent challenges in this domain (Hostettler et al., 2019; Zhu et al., 2019). Consequently, the development of novel therapeutic strategies is critically important to enhance ICH prognosis and reduce the detrimental effects associated with SBI. Factors such as oxidative stress, neuronal damage, inflammation, and increased thrombin due to hemorrhage contribute to SBI (Wang et al., 2018; Shao et al., 2019). These processes result in compromised blood-brain barrier (BBB) integrity, brain edema, and neuronal death (Wang et al., 2018). An increasing number of studies have shown that elevated thrombin levels after ICH will affect brain injury through multiple mechanisms (Lee et al., 1997; Caliaperumal et al., 2014; Wan et al., 2016). We expect to improve the understanding of SBI and search for more potential therapeutic targets by discussing the mechanisms of how thrombin affects SBI after ICH.

Thrombin, a multifunctional serine protease, plays a pivotal role as an effector protease within the blood coagulation system (Di Cera, 2008). Study indicates that post-ICH, thrombin levels around hematomas increase initially at 12 h, peak at 48 h, and stay high up to 72–108 h (Hui et al., 2013). Plasma thrombin-antithrombin (TAT) levels rise on day one and decline over time, remaining notably higher than in non-ICH individuals despite a significant decrease by day four (Wu et al., 2006). Additionally, both animal and human brain tissue studies have established a strong link between thrombin levels, brain edema, and brain cell apoptosis (Striggow et al., 2000; Wu et al., 2006). Thrombin, apart from its role in hemostasis (Danckwardt et al., 2013), exerts regulatory control over brain cell apoptosis and viability, neuroinflammatory processes, and BBB permeability through the activation of protease-activated receptors (PARs) (Zhang et al., 2010; Machida et al., 2015).

PARs belong to the G-protein-coupled receptor family (Heuberger and Schuepbach, 2019). As high-affinity thrombin receptors, PAR-1 and PAR-3 can be activated at lower concentrations, while PAR-4, as a low-affinity thrombin receptor, can only be activated at higher thrombin concentration (Coughlin, 1999; Ossovskaya and Bunnett, 2004). In addition to thrombin concentration, PAR activation is also influenced by PAR location (Danckwardt et al., 2013). The activation of PAR1 through thrombin-mediated mechanisms initiates classical tethered ligand activation, resulting in proinflammatory signaling and heightened endothelial permeability (Alberelli and De Candia, 2014). Conversely, other proteases cleave PAR1 at distinct locations, activating biased tethered ligands (Zhao et al., 2014). For instance, activated protein C (APC), triggered by thrombin-bound thrombomodulin in the endothelium, activates PAR1 at a nonclassical site, leading to anti-inflammatory effects and protection of the endothelial barrier (Han and Nieman, 2020). C4a, released by complement 4 (C4) during system activation, serves as an untethered ligand for PAR1 and PAR4 receptors, directly activating them and increasing endothelial permeability via the PAR1 pathway (Barnum, 2015; Wang et al., 2017). Depending on the variables regulating PAR activation, thrombin may exert dual effects on cells by contributing to anti-inflammatory and pro-inflammatory processes, modulating endothelial integrity and permeability, and affecting neuron viability (Alberelli and De Candia, 2014).

Consequently, this review provided a comprehensive overview of the specific molecular mechanisms underlying thrombin following ICH and its impact on various cells. Furthermore, we elucidated the implications of diverse thrombin concentrations on ICH and the potential therapeutic applications of thrombin inhibitor intervention.

## 2 Materials and methods

### 2.1 Information sources and search strategies

A comprehensive search of PubMed and Web of Science databases was conducted to identify relevant studies. The search was carried out from database inception to 30 November 2023. The search strategy used the following generic terms as search terms: “intracerebral hemorrhage,” “cerebral hemorrhage,” and “thrombin.” For example, the detailed search strategy for PubMed is as follows: ((intracerebral hemorrhage) or (cerebral hemorrhage)) and (thrombin). Further references were identified from included publications or available reviews.

### 2.2 Inclusion and exclusion criteria

Inclusion criteria were as follows: 1) studies investigating the mechanisms of intracerebral hemorrhage and thrombin, 2) experimental models in animal and/or cell culture, and 3) journal articles only.

Exclusion criteria were as follows: 1) non-spontaneous intracerebral hemorrhage or other diseases combined with intracerebral hemorrhage; 2) review; and 3) no thrombin involved.

### 2.3 Risk of bias assessment

The assessment of the quality of the animal studies included in this research was conducted using the risk of bias tool developed by the Systematic Review Centre for Laboratory Animal Experimentation (SYRCLE), which is based on the Cochrane RoB tool and modified to address specific biases in animal intervention studies (Hooijmans et al., 2014). Previous studies have demonstrated the efficacy of SYRCLE’s risk of bias tool for evaluating bias in animal studies (Zeng et al., 2015; Ahmed et al., 2022; Al-Masawa et al., 2022; Suresh et al., 2022). The tool contains selection bias (sequence generation, baseline characteristics, and allocation concealment), performance bias (random housing and blinding), detection bias (random outcome assessment and blinding), attrition bias (incomplete outcome data), reporting bias (selective outcome reporting), and other sources of bias. Two authors performed an independent quality assessment, and each methodological bias in the included animal studies was rated as “low risk,” “high risk,” or “unclear risk”.

## 3 Results

### 3.1 Study selection

**FIGURE 1:**
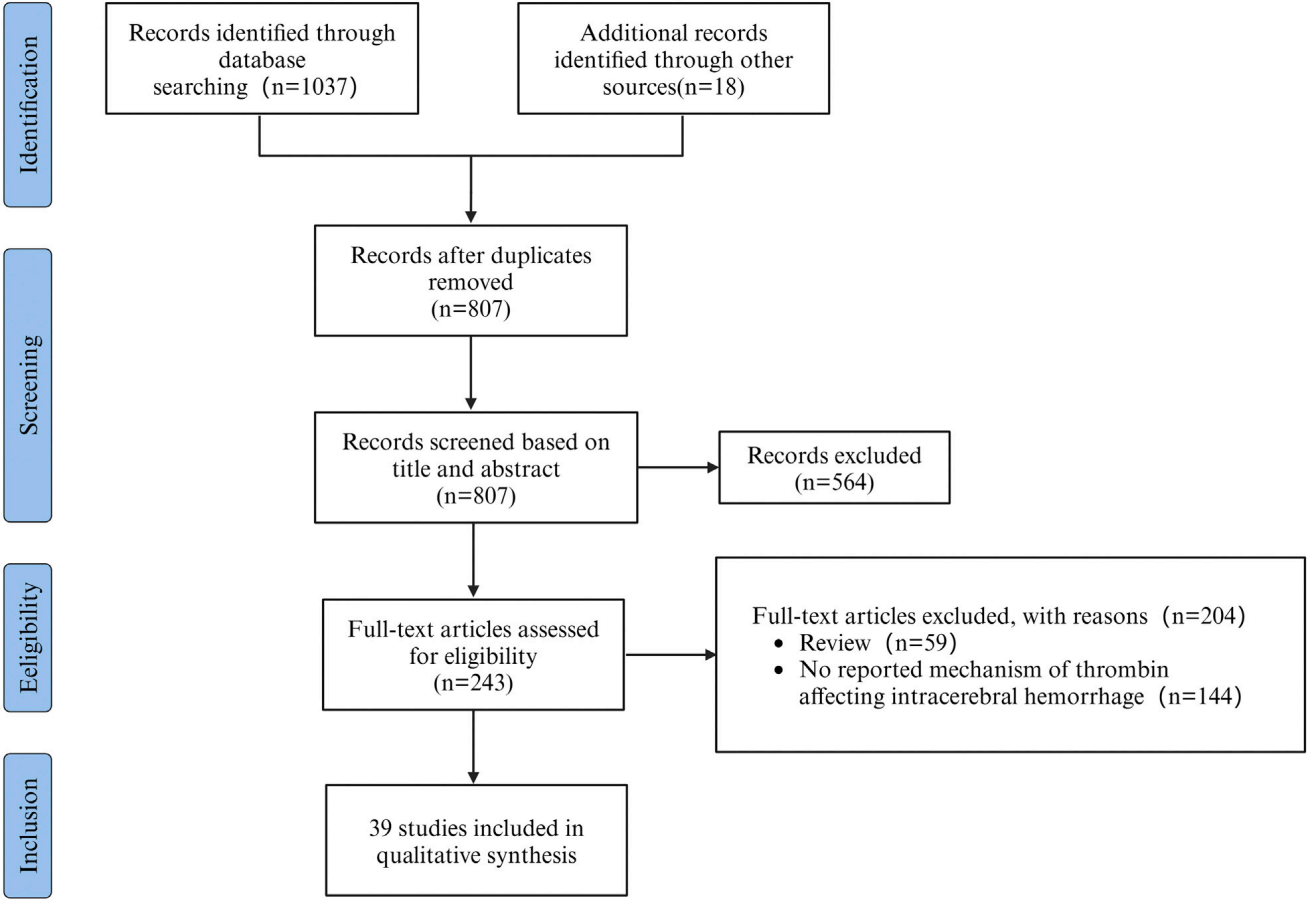
PRISMA flowchart outlining the process of retrieving and filtering articles, with n indicating the number of articles selected.

The aim of this review was to assess the potential mechanisms of thrombin influencing SBI after ICH. A total of 1037 articles were retrieved from literature databases and 18 articles were retrieved from other sources. Other sources were acquired through manual examination of the reference lists of the incorporated articles and relevant reviews. After removing 248 duplicates, 807 potentially relevant articles were evaluated. Subsequently, 564 articles were excluded after the evaluation of titles and abstracts. Among the remaining 243, 59 reviews were excluded, and 144 studies were excluded by screening the full text for failures to report mechanisms of thrombin affecting ICH. Therefore, 39 studies were included in the research. As shown in the flowchart (Figure 1). The data and characteristics of the included studies are shown in Table 1.

**TABLE 1:** The included articles’ data and characteristics.

Article	Study design	ICH models	Thrombin dose	Time	Year
Wu et al. (2010)	*In vivo* study	The Autologous blood model	-	2h/3h/6h/10h/12h/1d/2d/5d	2010
Wan et al. (2016)	*In vivo* study	The Autologous blood model	-	4h/1d/3d/7d	2016
Noda et al. (2020)	*In vitro* study	Thrombin stimulation	0,30,100,300U/ml	24h/48h/72 h	2020
Lee et al. (1997)	*In vivo* and *vitro* study	Thrombin injection/Thrombin stimulation	0,10,100 U/ml	1h/24 h	1997
Gong et al. (2005)	*In vivo* study	Thrombin injection	5U/animal	1d/3d/5d/7d/14d	2005
Nakamura et al. (2005)	*In vivo* study	Thrombin injection	1U/animal	24 h	2005
Li et al. (2019)	*In vivo* study	The type IV-S collagenase model	-	7d/14d/21d/28d	2019
Fujimoto et al. (2008)	*In vitro* study	Thrombin stimulation	30,100 U/ml	30/60/90/120/150/180min	2008
Xue and Del Bigio (2001)	*In vivo* study	Thrombin injection	2.5,25U/animal	300 h	2001
Figueroa et al. (1998)	*In vivo* study	Thrombin injection	0,1,5 8U/animal	24 h	1998
Hu et al. (2011)	*In vivo* and *vitro* study	Thrombin stimulation/The Autologous blood model	3U/animal	1d/3d/7d	2011
			0,3,5U/ml		
Hu et al. (2016)	*In vivo* and *vitro* study	Thrombin injection/Thrombin stimulation	1U/animal	4d	2016
			0,3,5 U/ml		
Hu et al. (2019a)	*In vivo* study	The Autologous blood model	1U/animal	3d/7d/14d	2019
Cui et al. (2020)	*In vivo* and *vitro* study	Thrombin injection/The Autologous blood model	1U/animal	3d/7d/14d	2020
				6h/48 h	
Zhou et al. (2012)	*In vivo* study	The Autologous blood model	1U/animal	3d/7d/14d	2012
Hu et al. (2019b)	*In vitro* study	Thrombin stimulation	0.5,1U/ml	-	2019
Brailoiu et al. (2017)	*In vitro* study	Thrombin stimulation	0.1,0.5,1U/ml	-	2017
Liu et al. (2010)	*In vivo* study	Thrombin injection	20U/animal	1d/7d/14d	2010
Machida et al. (2015)	*In vitro* study	Thrombin stimulation	1,3,10U/ml	24 h	2015
Xue et al. (2006)	*In vivo* and *vitro* study	Thrombin stimulation/The Autologous blood model	1,2,4,8,10 U/ml	24h/48 h	2006
			2 U/animal		
Kawakita et al. (2006)	*In vivo* study	Thrombin injection	3,10U/animal	12h/24h/72 h	2006
Gong et al. (2008)	*In vivo* study	The Autologous blood model/Thrombin injection	5U/animal	1h/24 h	2008
				1d/3d/28d	
Krenzlin et al. (2020)	*In vivo* study	The Autologous blood model/Silicone oil injection	-	24 h	2020
Wu et al. (2022)	*In vivo* and *vitro* study	Thrombin stimulation/The Autologous blood model	20U/ml	24h/72 h	2022
Caliaperumal et al. (2014)	*In vivo* study	Thrombin injection	1U/ml	7d/60d	2014
Donovan et al. (1997)	*In vitro* study	Thrombin stimulation	0,20,50,100,200U/ml	24 h	1997
Fujimoto et al. (2006)	*In vitro* study	Thrombin stimulation	10,30,100,300U/ml	24h/48h/72 h	2006
Ohnishi et al. (2007)	*In vivo* study	The type IV collagenase model	-	3d	2007
				4h/8h/24h/48 h	
Bao et al. (2017)	*In vitro* study	Thrombin stimulation	10,50,100U	0h/1h/6h/12h/24h/48 h	2017
Gingrich et al. (2000)	*In vitro* study	Thrombin stimulation	3,6,7U/ml	-	2000
García et al. (2015)	*In vitro* study	Thrombin stimulation	0.1,0.5,1,10 U/ml	1h/100 h	2015
Donovan and Cunningham (1998)	*In vitro* study	Thrombin stimulation	0.2,1,10,20,40,200U/ml	8h/12h/16h/20h/24h/72 h	1998
Sun et al. (2009)	*In vivo* study	The Autologous blood model	-	6h/24h/48h/72 h	2009
Nagatsuna et al. (2005)	*In vivo* study	The type IV collagenase model	-	24h/72 h	2005
Zhou et al. (2011)	*In vivo* study	Thrombin injection/The Autologous blood model	10U	24 h	2011
Ye et al. (2023)	*In vivo* study	Thrombin injection	0.5U	4 h	2023
Hijioka et al. (2020)	*In vivo* and vitro study	Thrombin stimulation/The type VII collagenase model	0,10,30,50U/ml	12h/24 h	2020
Chao et al. (2023)	*In vivo* and vitro study	Thrombin stimulation/The Autologous blood model	-	6h/24h/72 h	2023
Mu et al. (2017)	*In vitro* study	Thrombin stimulation	20U/ml	24 h	2017

### 3.2 Risk of bias of included animal studies

**FIGURE 2:**
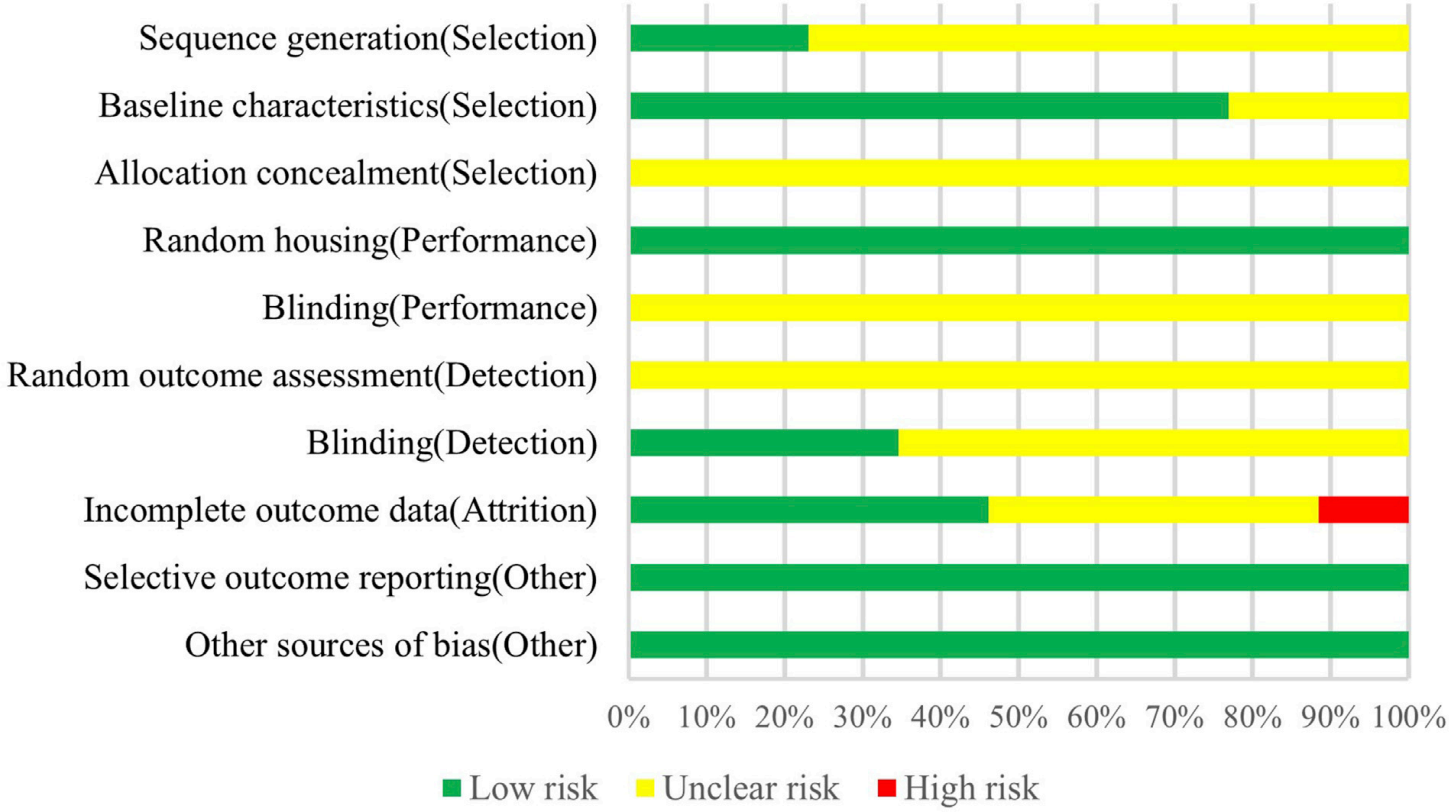
Risk of bias assessment of included animal studies.

According to the evaluation of SYRCLE’s ROB tool, among the 26 animal experimental studies included, 6 described random sequence generation (Zhou et al., 2012; Caliaperumal et al., 2014; Hu et al., 2019a; Li et al., 2019; Cui et al., 2020; Ye et al., 2023). Only 6 studies reported incomplete data on baseline characteristics (Gong et al., 2005; Xue et al., 2006; Zhou et al., 2011; Zhou et al., 2012; Wan et al., 2016; Chao et al., 2023), and all the remaining studies comprehensively reported baseline characteristics of the animals, including animal breed, age, weight, and sex. No studies reported information on allocation concealment and blinding of animal interventions by investigators or animal breeders. None of the studies could assess exact risk with respect to randomization for outcome assessment. In 9 studies (Xue and Del Bigio, 2001; Gong et al., 2005; Xue et al., 2006; Caliaperumal et al., 2014; Hu et al., 2016; Hu et al., 2019a; Li et al., 2019; Krenzlin et al., 2020; Ye et al., 2023), outcome assessors were blinded during the analysis. 12 studies fully accounted for incomplete outcome data (Figueroa et al., 1998; Xue and Del Bigio, 2001; Nagatsuna et al., 2005; Nakamura et al., 2005; Kawakita et al., 2006; Sun et al., 2009; Zhou et al., 2011; Caliaperumal et al., 2014; Li et al., 2019; Cui et al., 2020; Hijioka et al., 2020; Ye et al., 2023), 3 studies did not report complete outcome data (Lee et al., 1997; Gong et al., 2005; Zhou et al., 2012), and others had unclear risks on this item. Animal placement was randomized in all studies, and there was no selective outcome reporting and other sources of bias (As shown in Figure 2).

### 3.3 The molecular mechanism of thrombin in ICH-induced brain injury and repair

**FIGURE 3:**
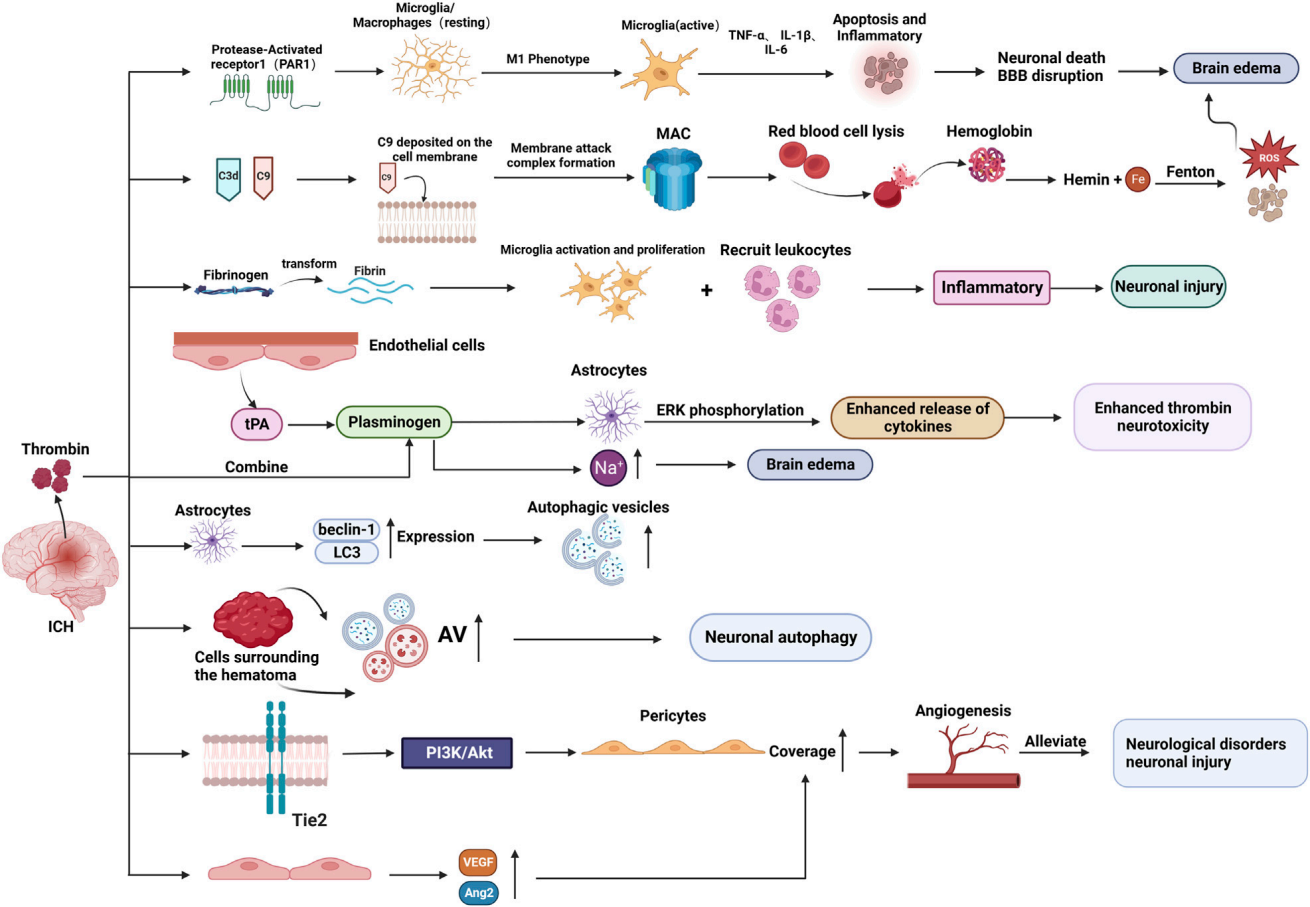
The molecular mechanism of thrombin involved in brain injury and repair after ICH. (BBB: blood-brain-barrier; MAC: membrane attack complex; AV: autophagic vacuoles; tPA: tissue plasminogen activator.) Created with BioRender.com.

After ICH, thrombin promotes SBI (via several pathways that exacerbate brain edema and neuronal damage) and could potentially also facilitate the repair process. The molecular mechanism of thrombin’s role in ICH injury and repair is detailed in the following sections (As shown in Figure 3).

#### 3.3.1 Thrombin-induced inflammatory response in ICH

Following ICH, the brain experiences an inflammatory response which causes infiltrating leukocytes and activated microglia to release cytotoxic mediators, leading to SBI and brain edema formation (Gong et al., 2000; Wu et al., 2010).

By activating PAR-1, thrombin increases the inflammatory response, which may result in excessive microglia/macrophage activation (Wan et al., 2016). The release of pro-inflammatory cytokines, including tumor necrosis factor-alpha (TNF-α) and interleukin-1beta (IL-1β) (Golderman et al., 2022), is enhanced by the microglia/macrophage bias toward a skewed M1 phenotype which is modulated in part to cause microglia/macrophage-mediated inflammatory brain injury (Hu et al., 2015). This process may worsen neuronal death and BBB disruption, as well as brain edema and neurological impairments after ICH (Wan et al., 2016).

Thrombin receptors PAR-1 and PAR-4 are expressed in neutrophils, and it was discovered that thrombin triggers neutrophils to express pro-inflammatory and anti-inflammatory phenotypes (Fu et al., 2015). The coexistence of cortical striatal cultures during thrombin triggering enhanced the pro-inflammatory response and decreased the anti-inflammatory response of HL60 neutrophils (Noda et al., 2020).

#### 3.3.2 Thrombin-induced iron deposition

The heme degradation process results in the accumulation of ferrous iron, which leads to microglia activation, neutrophil infiltration, and the production of reactive oxygen species (ROS), consequently mediating the intimations of inflammatory responses and neuronal death, thus leading to SBI (Wu et al., 2011). Additionally, iron combined with ferritin can induce neuronal death.

Furthermore, the BBB and complement cascade reaction are disrupted and activated, respectively, when thrombin is released following an ICH episode (Lee et al., 1997; Hua et al., 2007; Kearns et al., 2021). Thrombin mainly activates complement components C3d and C9 (Hua et al., 2000; Gong et al., 2005). The formation of membrane attack complexes (MACs) is indicated by the deposition of C9 on neuronal cell membranes after ICH (Hua et al., 2000). The hemolysis occurs when MACs develop. Hemoglobin is broken down to heme and iron once erythrocytes start to lyse, and the released iron catalyzes the Fenton reaction, resulting in oxidative stress and cell death, consequently causing neuronal death and aggravating brain edema (Wu et al., 2011; Babu et al., 2012; Kearns et al., 2021).

Transferrin (Tf) is a crucial iron carrier through the plasma to various tissues. Plasma contains two forms of transferrin: iron-bound (holo-Tf) and iron-free (apo-Tf) (Nakamura et al., 2005). The Holo-Tf has a high affinity for Tf receptors, which results in endocytosis and ferrous iron (a reduced form of iron) release during receptor interactions (Templeton and Liu, 2003). In the brain, the Tf receptor is involved in iron transit between the blood and the brain, as it is abundantly present in endothelial cells constituting the BBB (Broadwell et al., 1996). Thrombin activates Tf receptors on neurons, and parenchymal cells take up the transferrin-bound iron (Hua et al., 2003). Consequently, intracellular iron levels increase, leading to free radical production, oxidative damage, cell death, and, eventually, brain edema (Tampo et al., 2003).

#### 3.3.3 Plasminogen enhances the cytotoxicity of thrombin

Following ICH, thrombin converts fibrinogen into fibrin to achieve hemostasis. However, according to some studies, fibrin could increase nerve damage by inducing microglia to proliferate and recruit leukocytes to enhance inflammation (Paul et al., 2007; Li et al., 2019). Furthermore, fibrin contributes to edema formation. The levels of fibrinogen in the brain exhibit a notable increase during the advanced stages of ICH, and the modulation of fibrinogen could potentially play a role in the recuperation process of ICH (Ryu et al., 2015). Plasmin is the other serine protease involved in fibrin dissolution. The brain endothelium produces tissue plasminogen activator (tPA), which converts plasminogen (the precursor protein of plasmin) to plasmin.

Although plasminogen alone does not cause significant neuronal injury, its combined use with thrombin can lead to. When used in combination, plasminogen enhances the neurotoxicity of thrombin (30 U/mL) in the cerebral cortex and causes cortical damage (Fujimoto et al., 2008). Plasminogen activity and ERK phosphorylation in astrocytes may mediate this process, and plasminogen activation of astrocytes may lead to increased cytokine production that makes neurons susceptible to thrombin (Xue and Del Bigio, 2001; Fujimoto et al., 2008).

Furthermore, a comparison of co-infusion of plasminogen activator with thrombin with the administration of thrombin alone showed higher sodium accumulation in the brain, which promoted brain edema development (Menzies et al., 1993; Figueroa et al., 1998). These findings imply that the plasminogen/plasmin system increases thrombin neurotoxicity and promotes brain edema formation.

#### 3.3.4 Thrombin induces autophagy in ICH

Autophagy is a cellular degradation process involving isolating cellular proteins and organelles in autophagosomes (double-membrane vesicles), which are subsequently transported to lysosome and digested by lysosomal hydrolases (Wang and Klionsky, 2003).

*In vivo*, thrombin stimulation increased beclin-1 and light chain 3 (LC3) expression in rat astrocytes and stimulated the development of autophagosomes within astrocytes. Moreover, *in vitro*, thrombin boosted LC3-II levels and the amount of MDC-labeled autophagic vesicles in cultured astrocytes. These findings imply that thrombin induces autophagy in both the brain and cultured astrocytes (Hu et al., 2011; Hu et al., 2016).

A recent study discovered that by increasing the amount of autophagic vacuoles (AVs; both autophagosomes and autolysosomes) in the cells surrounding the hematoma, thrombin stimulates autophagy in neurons around the hematoma in the brains of ICH patients (Wu et al., 2019). Another study discovered that thrombin could activate autophagy and aggravate brain injury by increasing LC3-I to LC3-II conversion and histone D levels and promoting AV formation in neurons following its injection into the rat brain (Adhami et al., 2006).

Paradoxically, this process can have dual effects: promoting neuronal survival and causing neuronal damage or death. Therefore, autophagy after ICH may be beneficial or detrimental (Niu et al., 2017; Wu et al., 2019).

#### 3.3.5 Thrombin promotes angiogenesis in ICH

Angiogenesis is an essential endogenous brain self-repair process for neurological recovery after ICH (Cui et al., 2022). According to recent studies, low thrombin doses administered in the ICH rat model increase pericyte coverage by activating the angiopoietin receptor (Tie2) and downstream PI3K/Akt signaling, and the increased pericyte coverage subsequently promotes the maturation and stabilization of new vessels, alleviating neurological dysfunction and neuronal injury post-ICH (Hu et al., 2019a).

Another study discovered that large amounts of thrombin were released after ICH, and thrombin upregulated microRNA-24-1-5p (miR-24), suppressing the PHD1 protein expression (Cui et al., 2020). The PHD1 is a key prolyl hydroxylase of hypoxia-inducible factor-1α (HIF-1α), and a decrease in PHD1 correspondingly triggers a decrease in HIF-1α degradation (Cui et al., 2020). The HIF-1α is a nuclear transcription factor and hub mediator of angiogenesis; therefore, miR-24 promotes thrombin-induced angiogenesis by targeting PHD1 (Kuschel et al., 2012; Cui et al., 2020). Angiogenesis essentially facilitates brain recovery and functional improvement by increasing the local blood and oxygen supply to the brain injury, promoting oxygen and metabolite exchange, and removing toxic substances (Zhou et al., 2012).

By upregulating vascular endothelial growth factor (VEGF) and angiopoietin-2 (Ang-2) levels, thrombin activates quiescent brain endothelial cells and stimulates endothelial cell proliferation, migration, and new vessel formation, while it also upregulates Ang-1 levels to stabilize vascular integrity and shift neovascularization to maturation (Zhou et al., 2012). Additionally, thrombin stimulates angiogenesis in astrocytes by activating PAR-1 and p44/42 MAPK in astrocytes and upregulating VEGF release (Hu et al., 2019b).

### 3.4 The effect of thrombin on various types of cells after ICH

**FIGURE 4:**
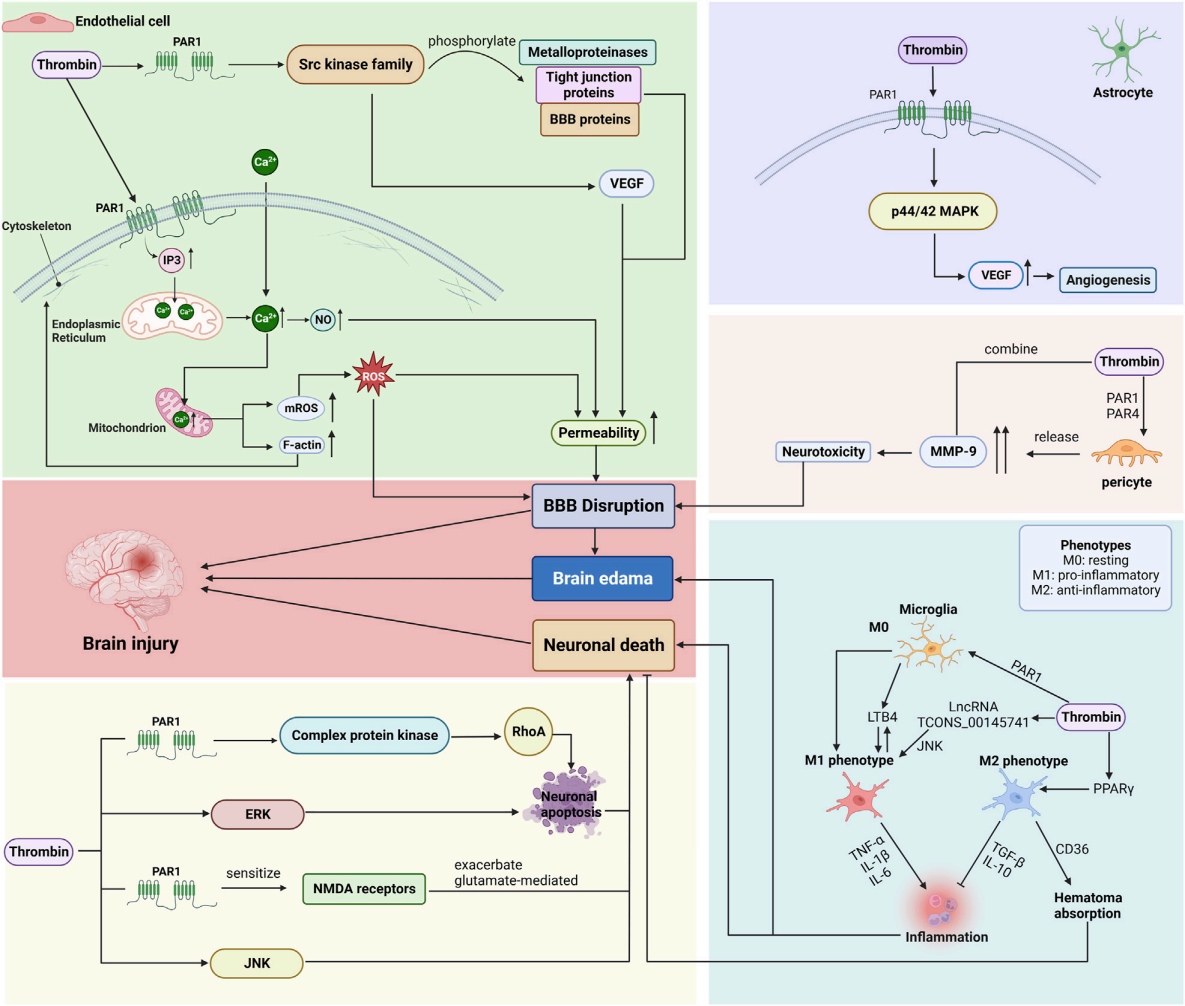
Mechanisms by which thrombin affects endothelial cells, astrocytes, pericytes, microglia, and neurons after ICH. (BBB: blood-brain barrier; PAR: protease-activated receptor; VEGF: vascular endothelial growth factor; ROS: reactive oxygen species; ERK: signal-regulated kinase; JNK: c-Jun N-terminal kinase.) Created with BioRender.com.

Following ICH, thrombin mainly affects neurons and microglia, as well as various types of cells that constitute the BBB (Zhou and Li, 2002; Wang et al., 2016). The brain microvascular endothelial cells (BMVECs), astrocytes, pericytes, and basement membranes make up most of the BBB. Endothelial cells form the capillary wall and are the primary BBB barrier. The astrocyte end-foot wraps around the BMVECs, the cell-secreted matrix proteins form the basement membrane, and the pericytes are embedded in the basement membrane of the glial cells and BMVECs (As shown in Figure 4; Table 2).

**TABLE 2:** Mechanism of thrombin in various cells after ICH.

Cell types	Animals/Cells	ICH models	Time	Thrombin dose	Mechanism	Outcomes	Refs
Endothelial cells	Rat brain microvascular endothelial cells	Thrombin stimulation	-	0.1,0.5,1U/ml	Increased NO and ROS	Increased BBB permeability and disrupted BBB function	Brailoiu et al. (2017)
Endothelial cells	Male Sprague-Dawley rats	Thrombin injection	1d/7d/14d	20U/animal	Activation of SRC kinase family members to induce decreased immune responsiveness of endothelial cells, resulting in endothelial cell damage or death	Increased BBB permeability and brain edema	Liu et al. (2010)
Endothelial cells	Male Sprague-Dawley rats	The Autologous blood model/Thrombin injection	3d/7d/14d	1U/animal	Upregulation of miR-24, which inhibited PHD1 protein expression, and the reduction of PHD1 triggered a decrease in HIF-1αdegradation	Angiogenesis	Cui et al. (2020)
Endothelial cells	Male Sprague-Dawley rats	The Autologous blood model/Thrombin injection	1d/3d/7d	1U/animal	Activation of quiescent brain endothelial cells and upregulation of VEGF and Ang-2 levels	Endothelial cell proliferation, migration and neointima formation	Zhou et al. (2012)
Astrocytes	Male Sprague-Dawley rats	Thrombin injection	1d/7d/14d	20U/animal	Reduced immune responsiveness of perivascular astrocytes	Increased BBB permeability, increased brain water content, and BBB destruction	Liu et al. (2010)
Astrocytes	Rat/mouse astrocytes	Thrombin stimulation	-	0.5,1U/ml	Stimulation of PAR-1 and p44/42 MAPK in astrocytes to upregulate VEGF release	Angiogenesis of astrocytes	Hu et al. (2019b)
Pericytes	Rat brain pericytes	Thrombin stimulation	24 h	1,3,10U/ml	Stimulation of high levels of MMP-9 release from pericytes	BBB dysfunction	Machida et al. (2015)
Pericytes	Male Sprague-Dawley rats	The Autologous blood model/Thrombin injection	1d/4d/7d	1U/animal	Activated Tie2 and downstream PI3K/Akt signaling to increase pericyte coverage	Promoted maturation and stabilization of neovascularization and alleviated neurological dysfunction and neuronal damage after ICH	Hu et al. (2019a)
Microglia	Male C57BL/6,wild-type (WT) mice, male PAR-1 knockout, (PAR-1 KO) mice	The Autologous blood model	1d/3d/7d	-	Regulation of microglia/macrophage polarization toward M1 phenotype to promote the release of inflammatory factors	Exacerbated neuronal death and brain edema post-ICH	Wan et al. (2016)
Microglia	Male Tmem119-EGFP mice	Thrombin injection	4 h	0.5U/animal	Stimulation of microglia	Microglia proliferation	Ye et al. (2023)
Microglia	Male C57BL/6J mice, Mouse microglia (BV2)	Thrombin stimulation/The type VII collagenase model	12h/24 h	0,10,30,50U/ml	promoted LTB4 secretion by microglia	Inflammatory nerve injury	Hijioka et al. (2020)
Microglia	Male C57BL/6 mice, Mouse microglia (BV2)	The Autologous blood model/Thrombin stimulation	6h/24h/72 h	-	Induction of M1 polarization in microglia	Inflammation	Chao et al. (2023)
Microglia	Mixed primary microglia cultures	Thrombin stimulation	24 h	20U/ml	Activation of microglia to express higher levels of CD36 protein and PPARγ mRNA to enhance microglia phagocytosis	Clearance of hematoma	Mu et al. (2017)
Neurons	Rat hippocampal neurons	Thrombin stimulation	24 h	0,20,50,100,200U/ml	Induction of complex kinase activity to activate RhoA activity	Neuronal apoptosis	Donovan et al. (1997)
Neurons	Cortico-striatal slices	Thrombin stimulation	24h/48h/72 h	10,30,100,300U/ml	Increased ERK phosphorylation and sustained signaling Src and PKC induced	Neuronal injury in the cerebral cortex and striatum	Fujimoto et al. (2006)
Neurons	Cortical neurons	Thrombin stimulation	0h/1h/6h/12h/24h/48 h	10,50,100U	Activation of JNK	Neuronal apoptosis	Bao et al. (2017)
Neurons	Rat hippocampal neurons	Thrombin stimulation	-	3,6,7U/ml	Activation of PAR-1 to enhance the NMDA receptors sensitivity	Neuronal death	Gingrich et al. (2000)

#### 3.4.1 Endothelial cells

BMVECs are an essential component of the BBB, as the complex tight junctions between adjacent endothelial cells form a physical barrier that forces most molecular traffic through the transcytosis/transport protein route across the BBB (Persidsky et al., 2006).

Thrombin acts primarily on PAR-1, triggering an increase in inositol 1,4,5-trisphosphate (IP3), which interacts with the IP3 receptor to release Calcium (Ca^2+^) from the endoplasmic reticulum (ER), and induces an influx of Ca^2+^ from outside the cell, elevating the cytosolic Ca^2+^ concentration in BMVEC (Brailoiu et al., 2017). Ca^2+^ is a critical second messenger regulating barrier function, and the endothelial Ca^2+^ concentration determines paracellular permeability (De Bock et al., 2013). The increased Ca^2+^ concentration promotes nitric oxide (NO) formation, which causes cytoskeletal alterations (increased F-actin stress fiber formation) and disruption of tight junctions, leading to increased permeability and barrier dysfunction (Fleming et al., 1997; Wang et al., 2015).

Furthermore, thrombin-induced elevation of Ca^2+^ is transmitted to the mitochondria, increasing the Ca^2+^ concentration in mitochondria and thereby triggering mitochondrial reactive oxygen species (mROS) generation (Camello-Almaraz et al., 2006). Endothelial dysfunction is associated with ROS production as ROS accumulation induces oxidative stress, which is involved in several cellular processes, including inflammatory response, apoptosis, autophagy, and SBI caused by disruption of the BBB (Chen et al., 2022; Zhang et al., 2022).

Research has revealed that thrombin exhibits binding capabilities towards PAR, thereby initiating the activation of various members belonging to the complex kinase family, commonly referred to as the src kinase family (Liu et al., 2010). Members of the src family of kinases can influence changes in BBB permeability and brain edema by phosphorylating metalloproteinases, tight junction proteins, and other BBB proteins and by increasing VEGF induction (Guerrero et al., 2004; Liu et al., 2010). The VEGF is a vascular endothelial cell-specific mitogen that induces endothelial cell proliferation and promotes increased vascular extravasation, increasing BBB permeability and brain edema.

Therefore, thrombin plays a role in disrupting the BBB function by regulating the activity of BMVECs via several mechanisms. Injury to the BBB can cause secondary damage in ICH and promote edema formation or development after ICH (Zheng et al., 2016; Noda et al., 2020).

#### 3.4.2 Astrocytes

Astrocytes, a vital component of the BBB, are glial cells that wrap around 99% of the BBB endothelium, interact with endothelial cells, and contribute to the formation and maintenance of tight junctions (Persidsky et al., 2006).

Thrombin lowered the BMVEC and perivascular astrocyte immunoreactivity, implying cell injury or death, which amplifies BBB permeability, increasing brain water content and BBB destruction (Liu et al., 2010). Another study has found that thrombin mediated VEGF secretion via the PAR-1 and p44/42 MAPK pathways (Hu et al., 2019b). The VEGF release stimulated angiogenesis in astrocytes (Hu et al., 2019b).

#### 3.4.3 Pericytes

Pericytes are flat, undifferentiated, contractile connective tissue cells that develop around the capillary wall. Pericytes are closely associated with endothelial cells, and their absence results in endothelial hyperplasia and abnormal vascular morphogenesis in the brain (Persidsky et al., 2006). Pericytes also play a vital role in maintaining the structural integrity of the BBB.

After ICH, the levels of matrix metalloproteinase (MMP) in the brain tissue increase, promoting neuronal death, leading to BBB breakdown, consequently promoting brain edema formation and ultimately leading to brain hemorrhage-induced secondary damage (Florczak-Rzepka et al., 2012; Dang et al., 2017). During the acute ICH phase, thrombin levels in BBB cells are elevated, and thrombin acts through PAR-1 and PAR-4 to stimulate the release of high levels of MMP-9 from pericytes (Machida et al., 2015). Thrombin and MMP-9 are synergistically toxic, and their interaction increases neurotoxicity, eventually leading to further BBB dysfunction (Kawakita et al., 2006; Xue et al., 2006; Machida et al., 2015).

#### 3.4.4 Microglia

Microglia, as resident macrophages in the brain, play a crucial role in maintaining homeostasis within the central nervous system (CNS) (Ginhoux et al., 2010). The activation and polarization of microglia/macrophages have significant implications for SBI. Within 6 h after ICH, both M1 and M2 phenotypes experience an increase, although the elevation of M1 predominates (Liesz et al., 2011; Tschoe et al., 2020). By the 3rd day, the M1 phenotype reaches its peak and begins to decline, accompanied by a rise in the proportion of M2 observed in the perihematoma region (Wan et al., 2016; Lan et al., 2017). At the 14th day, the number of M2 microglia/macrophages reaches its peak and surpasses that of M1 (Tschoe et al., 2020). Microglia have the potential to transition between the M1 and M2 phenotypes, showcasing a significant level of plasticity (Hu et al., 2015; Lan et al., 2017).

The involvement of thrombin in the activation and polarization of microglia/macrophages after ICH has been confirmed. The activation of PAR-1 by thrombin regulates the polarization of microglia towards the M1 phenotype (Wan et al., 2016; Chao et al., 2023). Additionally, thrombin induces the differentiation of microglia into M1 phenotype by upregulating the expression of LncRNA TCONS_00145741 and activating the JNK MAPK pathway (Wu et al., 2022). Furthermore, thrombin production has been shown to stimulate the proliferation of microglia and induce the secretion of leukotriene B4 (LTB4) by these microglia (Hijioka et al., 2020; Ye et al., 2023). Consequently, the activation of microglia by LTB4 results in the generation of proinflammatory factors and the infiltration of neutrophils into hematomas, thereby exacerbating the inflammatory injury (Hijioka et al., 2020). Additionally, thrombin induces the upregulation of PPARγ levels, resulting in heightened expression of the scavenger receptor cluster of differentiation 36 (CD36) on microglial surfaces, thus facilitating the differentiation of microglia towards the M2 phenotype (Fang et al., 2014). The upregulated CD36 receptor enhances the phagocytic activity of activated microglia, thereby promoting the resorption of hematoma through the phagocytosis of erythrocytes (Mu et al., 2017).

In conclusion, thrombin can induce the activation of microglia (both M1 phenotype and M2 phenotype) and the induction bias may be influenced by variations in time and concentration (Wu et al., 2022). The regulation of phenotypic equilibrium could emerge as a novel therapeutic objective for ICH.

#### 3.4.5 Neurons

Neuronal damage post-ICH can cause severe behavioral dysfunction and exacerbate SBI (Gong et al., 2008). Early neuronal damage is associated with thrombin development. Cell death, severe dendritic damage of nearby striatal neurons, and eventual neuronal atrophy are all caused by thrombin perfusion (Caliaperumal et al., 2014). In addition, thrombin induces neuronal damage via several pathways.

First, thrombin activates the complex protein kinase via PAR-1 activation, triggering RhoA activation, which induces neuronal apoptosis (Donovan et al., 1997). Secondly, thrombin induces apoptosis of perihematomal neurons by activating several intracellular signaling enzymes. Mitogen-activated protein kinase (MAPK) signaling is critical for thrombin-induced neuronal death (Fujimoto et al., 2007). Some members of the MAPK family include extracellular signal-regulated kinase (ERK) and c-Jun N-terminal kinase (JNK). Inhibition of ERK was reported to significantly reduce thrombin-induced neuronal death (Fujimoto et al., 2006). On the other hand, high thrombin concentrations (100 U) activated JNK in primary cultured cortical neurons in a time-dependent manner, and direct thrombin stimulation-induced neuronal injury was partially prevented by the JNK pathway inhibitor SP600125, implying that thrombin induces neuronal apoptosis via JNK activation (Ohnishi et al., 2007; Bao et al., 2017). Additionally, thrombin can aggravate glutamate-mediated neuronal death by activating PAR-1 to enhance the NMDA receptor sensitivity (Gingrich et al., 2000; Zhou and Li, 2002).

### 3.5 Effect of thrombin concentration in ICH

According to researches, 0.01 U/ml of thrombin is protective against several causes of neuronal cell injuries, including glucose deprivation, hypoglycemia, and ROS (Striggow et al., 2000; Krenzlin et al., 2020). On the other hand, thrombin >10 U/ml exhibits cytotoxicity and kills neuronal cells, resulting in cellular damage and upregulation of TNF-α, with subsequent worsening of brain edema and neurological deficits after ICH (Donovan and Cunningham, 1998; Striggow et al., 2000; García et al., 2015). Another study discovered that treatment of hippocampal neurons with thrombin concentrations >150 U/ml (750 nm) resulted in a rapid and substantial increase in RhoA activity, leading to neuronal apoptosis, whereas neurons treated at lower thrombin concentrations exhibited a relatively less significant elevation in RhoA activity (Donovan et al., 1997). Treatment of cortico-striatal sections with thrombin concentrations >100 U/ml for over 24 h induced neuronal injury, with the extent of injury increasing radically with the increase in both thrombin concentration and duration of treatment (Fujimoto et al., 2006). Furthermore, treatment of cortical neurons with thrombin concentrations >50 U significantly increased neuronal apoptosis rate, which increased substantially with increasing thrombin dose (Bao et al., 2017). When administered at 20 U/animal, thrombin induced endothelial cell injury or death and ultimately decreased BBB permeability and increased brain edema, whereas it promoted endothelial cell proliferation, migration, neointima formation, and angiogenesis when administered at 1 U/animal (Liu et al., 2010; Zhou et al., 2012; Cui et al., 2020). Following treatment with thrombin (10 U/mL), brain pericytes exhibited extremely high levels of MMP-9 release, resulting in BBB injury, whereas following treatment with 1 U/mL and 3 U/mL of thrombin, brain pericytes showed substantially lower levels of MMP-9 release compared to the 10 U/mL dose (Machida et al., 2015).

The above research findings confirm that thrombin-induced brain injury is concentration-dependent, with high concentrations causing BBB injury, brain edema, and neuronal apoptosis, and low concentrations promoting neuronal growth and branching, improving neuronal viability, and preventing excitotoxic injury (Striggow et al., 2000; García et al., 2015). In an *in vitro* experiment, cortical neuronal cells were stimulated with different concentrations of thrombin and argatroban (a direct thrombin inhibitor), and neuronal survivability was assessed using the MTT assay. The results showed that lower concentration of thrombin (1 nM) exhibited comparable levels of neuroprotection as micromolar concentrations of argatroban (García et al., 2015).

### 3.6 Thrombin therapy

With the mechanism of secondary injury after ICH having been studied extensively, the comprehension of the effect of thrombin on secondary injury post-ICH has gradually improved. Thrombin inhibitors can improve thrombin-induced injury post-ICH by directly suppressing thrombin, which offers a novel way for future SBI treatment after ICH.

Hirudin is a potent, specific, natural direct thrombin inhibitor that binds directly to thrombin and prevents it from interacting with its substrate, inhibiting the conversion of fibrinogen to fibrin (Bichler and Fritz, 1991). According to an ICH mouse model, hirudin inhibited fibrin formation, reducing neuroinflammation and improving long-term outcomes (Li et al., 2019). Hirudin therapy reduced leukocyte accumulation in the brain and shifted microglia to an anti-inflammatory phenotype (Li et al., 2019). In other studies, hirudin was reported to alleviate thrombin-induced autophagy after ICH (Hu et al., 2011). Recombinant hirudin (r-Hirudin), a tight-binding specific thrombin inhibitor, prevents cytotoxicity in neurons, microglia, and astrocytes by blocking the induction of Aquaporin (AQP) 4 and 9, which are implicated in edema formation, thereby significantly reducing edema after ICH (Sun et al., 2009).

Argatroban is a small molecule, synthetic, direct, and competitive thrombin inhibitor. It rapidly and reversibly binds to the catalytic site of thrombin, preventing fibrin formation (McKeage and Plosker, 2001). In an ICH rat model, argatroban administration rapidly suppressed inflammatory cell infiltration within 24 h and reduced edema size to 25% within 72 h, contributing to improved prognosis (Nagatsuna et al., 2005; Zhou et al., 2011). Argatroban administered an hour post-ICH rapidly reduced the infiltration of polymorphonuclear neutrophils (PMNs), which produce free radicals that damage cellular functions, including neurons (Nagatsuna et al., 2005).

Administration of thrombin inhibitors following ICH has been shown to effectively mitigate neuroinflammation and brain edema, thereby enhancing prognosis. However, the comprehensive suppression of thrombin activity would yield adverse consequences due to the demonstrated neuroprotective and angiogenic properties associated with low levels of thrombin. Consequently, excessive utilization of thrombin inhibitors may exacerbate secondary injury and impede the prospects of long-term recuperation (Belur et al., 2013).

## 4 Conclusion

Thrombin is a critical component of the coagulation system that substantially impacts the secondary injury process after ICH. Following ICH, thrombin initiates an inflammatory cascade, characterized by the augmented activation of microglia/macrophages and the subsequent release of pro-inflammatory cytokines such as TNF-α and IL-1β (Wan et al., 2016). These cytokines contribute to a series of detrimental outcomes including compromise of BBB, development of brain edema, and overall neurological dysfunction (Hu et al., 2015). Additionally, thrombin is implicated in promoting iron deposition in the brain, predominantly via complement cascade activation (Hua et al., 2000). The iron-catalyzed Fenton reaction leads to oxidative stress-induced neuronal damage and aggravated brain edema (Babu et al., 2012). Furthermore, the interaction between thrombin and plasminogen intensifies neurotoxicity, further escalating neuronal injury and edema (Fujimoto et al., 2008). Interestingly, thrombin also plays a role in modulating autophagy within brain cells, a mechanism that may be beneficial or detrimental (Wu et al., 2019). Lastly, thrombin significantly contributes to the process of angiogenesis, which is crucial for neurological recuperation post-ICH (Cui et al., 2022). It activates molecular pathways that lead to enhanced pericyte coverage, vascular maturation, and stabilization, thereby aiding in the restoration of neurological function and mitigating neuronal damage (Hu et al., 2019a).

Following ICH, thrombin mainly affects neurons, microglia, and BBB components, including endothelial cells, pericytes, and astrocytes. Thrombin affects BMVECs by elevating cytoplasmic and mitochondrial Ca^2+^ levels and activating the SRC kinase family, thereby disrupting BBB function which leads to increased permeability and barrier dysfunction (Liu et al., 2010; Brailoiu et al., 2017; Zhang et al., 2022). In addition, thrombin has been observed to diminish the viability of BMVEC and astrocytes, while also intensifying BBB permeability (Liu et al., 2010). However, it also plays a role in facilitating the secretion of VEGF in astrocytes, thereby promoting angiogenesis (Hu et al., 2019b). Furthermore, thrombin activates MMP-9 release from pericytes through PAR-1 and PAR-4 pathways after ICH, intensifying neurotoxicity and compromising BBB integrity (Machida et al., 2015). Moreover, thrombin regulates microglia polarization, promoting M1 phenotype via PAR-1 activation and LncRNA TCONS_00145741/JNK MAPK pathway, while also stimulating microglia proliferation and LTB4 secretion, leading to inflammatory injury and neutrophil infiltration (Hijioka et al., 2020; Wu et al., 2022). Concurrently, thrombin fosters M2 phenotype differentiation through PPARγ upregulation and CD36 receptor enhancement on microglia, aiding hematoma resorption (Mu et al., 2017). Lastly, thrombin induces neuronal apoptosis by activating protein kinases through PAR-1, particularly MAPK pathways like ERK and JNK, and exacerbates glutamate-related neuronal death by increasing NMDA receptor sensitivity (Gingrich et al., 2000; Fujimoto et al., 2006; Bao et al., 2017).

However, thrombin release post-ICH is not exclusively an adverse consequence. Although high thrombin concentrations can damage neurons, promote inflammatory responses, destroy the BBB as well as promote the development and exacerbation of brain edema (Striggow et al., 2000), low thrombin concentrations can increase pericyte coverage, stimulate endothelial cells and astrocytes to upregulate VEGF, promote angiogenesis, protect neurons, and alleviate neurological dysfunction after ICH (García et al., 2015). The utilization of direct thrombin inhibitors, such as hirudin and argatroban, has demonstrated efficacy in enhancing SBI following ICH, consequently leading to improved prognosis (Nagatsuna et al., 2005; Li et al., 2019). However, it is important to consider that the excessive administration of thrombin inhibitors for SBI may amplify the neuroprotective and angiogenic characteristics associated with reduced levels of thrombin (Belur et al., 2013).

There are some limitations to this systematic review. First, the limited scope of the initial search mechanism, which only encompassed the PubMed and Web of Science databases, may have resulted in a sample size of included studies that was insufficient. Second, we employed the SYRCLE’s ROB tool for assessing bias risk in animal studies, yet these investigations frequently omitted crucial methodological specifics, including blinding of investigator manipulations, statistical results, and randomized animal grouping. Therefore, most of these studies were classified as low or unclear risk, which significantly affected the outcomes of our systematic evaluation. Furthermore, the omission of clinical trials within the scope of this study, coupled with the limited implementation of thrombin therapy in clinical settings, more related trials need to be conducted in the subsequent researches.

In conclusion, future research endeavors should concentrate on mitigating thrombin’s detrimental impact in ICH while amplifying its protective functions, offering novel perspectives and methodologies for clinical ICH therapy.

## Figures and Tables

**Table:** 

**Ang-2**	angiopoietin-2
**APC**	activated protein C
**AQP**	Aquaporins
**AVs**	autophagic vacuoles
**BBB**	blood-brain-barrier
**BMVEC**	brain microvascular endothelial cells
**C4**	complement 4
**Ca** ^**2+**^	Calcium
**CD36**	cluster of differentiation 36
**ER**	endoplasmic reticulum
**ERK**	extracellular signal-regulated kinase
**HIF-1α**	hypoxia-inducible factor-1α
**ICH**	Intracerebral hemorrhage
**IL-1β**	interleukin-1beta
**IP3**	inositol 1,4,5-trisphosphate
**JNK**	c-Jun N-terminal kinase
**LC3**	light chain 3
**LTB4**	leukotriene B4
**MAC**	membrane attack complex
**MAPK**	mitogen-activated protein kinase
**miR-24**	microRNA-24-1-5p
**MMP**	matrix metalloproteinase
**mROS**	mitochondrial reactive oxygen species
**NO**	nitric oxide
**PAR-1 KO**	PAR-1 knockout
**PARs**	protease-activating receptors
**PMNs**	polymorphonuclear neutrophils
**PPARγ**	peroxisome proliferator-activated receptor-gamma
**r-Hirudin**	recombinant hirudin
**ROS**	reactive oxygen species
**SBI**	secondary brain injury
**SYRCLE**	Systematic Review Centre for Laboratory Animal Experimentation
**TAT**	thrombin-antithrombin
**Tf**	Transferrin
**TGF-B**	transforming growth factor-beta
**TM**	thrombomodulin
**TNF-α**	tumor necrosis factor-alpha
**tPA**	tissue plasminogen activator
**VEGF**	vascular endothelial growth factor
**WT**	wild-type

## Data Availability

The original contributions presented in the study are included in the article/Supplementary material, further inquiries can be directed to the corresponding authors.

## References

[B1] Adhami F. Liao G. Morozov Y. M. Schloemer A. Schmithorst V. J. Lorenz J. N. (2006). Cerebral ischemia-hypoxia induces intravascular coagulation and autophagy. Am. J. Pathol. 169, 566–583. 10.2353/ajpath.2006.051066 16877357 PMC1780162

[B2] Ahmed M. Best L. M. Pereira C. F. Boileau I. Kloiber S. (2022). Effects of endocannabinoid system modulation on social behaviour: a systematic review of animal studies. Neurosci. Biobehav Rev. 138, 104680. 10.1016/j.neubiorev.2022.104680 35513169

[B3] Alberelli M. A. De Candia E. (2014). Functional role of protease activated receptors in vascular biology. Vasc. Pharmacol. 62, 72–81. 10.1016/j.vph.2014.06.001 24924409

[B4] Al-Masawa M. E. Alshawsh M. A. Ng C. Y. Ng A. M. H. Foo J. B. Vijakumaran U. (2022). Efficacy and safety of small extracellular vesicle interventions in wound healing and skin regeneration: a systematic review and meta-analysis of animal studies. Theranostics 12, 6455–6508. 10.7150/thno.73436 36185607 PMC9516230

[B5] An S. J. Kim T. J. Yoon B. W. (2017). Epidemiology, risk factors, and clinical features of intracerebral hemorrhage: an update. J. Stroke 19, 3–10. 10.5853/jos.2016.00864 28178408 PMC5307940

[B6] Babu R. Bagley J. H. Di C. Friedman A. H. Adamson C. (2012). Thrombin and hemin as central factors in the mechanisms of intracerebral hemorrhage-induced secondary brain injury and as potential targets for intervention. Neurosurg. Focus 32, E8. 10.3171/2012.1.FOCUS11366 22463118

[B7] Bao L. Zu J. He Q. Zhao H. Zhou S. Ye X. (2017). Thrombin-induced apoptosis in neurons through activation of c-Jun-N-terminal kinase. Toxicol. Mech. Methods 27, 18–23. 10.3109/15376516.2016.1172691 27841083

[B8] Barnum S. R. (2015). C4a: an anaphylatoxin in name only. J. Innate Immun. 7, 333–339. 10.1159/000371423 25659340 PMC6738802

[B9] Belur P. K. Chang J. J. He S. Emanuel B. A. Mack W. J. (2013). Emerging experimental therapies for intracerebral hemorrhage: targeting mechanisms of secondary brain injury. Neurosurg. Focus 34, E9. 10.3171/2013.2.FOCUS1317 23634928

[B10] Bichler J. Fritz H. (1991). Hirudin, a new therapeutic tool? Ann. Hematol. 63, 67–76. 10.1007/BF01707275 1912033

[B11] Brailoiu E. Shipsky M. M. Yan G. Abood M. E. Brailoiu G. C. (2017). Mechanisms of modulation of brain microvascular endothelial cells function by thrombin. Brain Res. 1657, 167–175. 10.1016/j.brainres.2016.12.011 27998795 PMC5350626

[B12] Broadwell R. D. Baker-Cairns B. J. Friden P. M. Oliver C. Villegas J. C. (1996). Transcytosis of protein through the mammalian cerebral epithelium and endothelium. III. Receptor-mediated transcytosis through the blood-brain barrier of blood-borne transferrin and antibody against the transferrin receptor. Exp. Neurol. 142, 47–65. 10.1006/exnr.1996.0178 8912898

[B13] Caliaperumal J. Brodie S. Ma Y. Colbourne F. (2014). Thrombin causes neuronal atrophy and acute but not chronic cell death. Can. J. Neurol. Sci. 41, 714–720. 10.1017/cjn.2014.105 25382279

[B14] Camello-Almaraz C. Gomez-Pinilla P. J. Pozo M. J. Camello P. J. (2006). Mitochondrial reactive oxygen species and Ca2+ signaling. Am. J. Physiol. Cell Physiol. 291, C1082–C1088. 10.1152/ajpcell.00217.2006 16760264

[B15] Chao C. Li Y. Li Q. Wu G. (2023). Inhibitory effect and mechanism of Rosiglitazone on M1 type polarization of central microglia in intracerebral hemorrhage mice based on JNK/STAT3 signaling pathway. Brain Behav. 13, e3275. 10.1002/brb3.3275 37837628 PMC10726784

[B16] Chen S. Li L. Peng C. Bian C. Ocak P. E. Zhang J. H. (2022). Targeting oxidative stress and inflammatory response for blood-brain barrier protection in intracerebral hemorrhage. Antioxid. Redox Signal 37, 115–134. 10.1089/ars.2021.0072 35383484

[B17] Coughlin S. R. (1999). How the protease thrombin talks to cells. Proc. Natl. Acad. Sci. U. S. A. 96, 11023–11027. 10.1073/pnas.96.20.11023 10500117 PMC34235

[B18] Cui H. Yang A. Zhou H. Wang Y. Luo J. Zhou J. (2020). Thrombin-induced miRNA-24-1-5p upregulation promotes angiogenesis by targeting prolyl hydroxylase domain 1 in intracerebral hemorrhagic rats. J. Neurosurg. 134, 1515–1526. 10.3171/2020.2.JNS193069 32413855

[B19] Cui Q. Zhang Y. Tian N. Yang J. Ya D. Xiang W. (2022). Leptin promotes angiogenesis via pericyte STAT3 pathway upon intracerebral hemorrhage. Cells 11, 2755. 10.3390/cells11172755 36078162 PMC9454866

[B20] Danckwardt S. Hentze M. W. Kulozik A. E. (2013). Pathologies at the nexus of blood coagulation and inflammation: thrombin in hemostasis, cancer, and beyond. J. Mol. Med. Berl. 91, 1257–1271. 10.1007/s00109-013-1074-5 23955016 PMC3825489

[B21] Dang B. Duan X. Wang Z. He W. Chen G. (2017). A therapeutic target of cerebral hemorrhagic stroke: matrix metalloproteinase- 9. Curr. Drug Targets 18, 1358–1366. 10.2174/1389450118666170427151657 28460607

[B22] De Bock M. Wang N. Decrock E. Bol M. Gadicherla A. K. Culot M. (2013). Endothelial calcium dynamics, connexin channels and blood-brain barrier function. Prog. Neurobiol. 108, 1–20. 10.1016/j.pneurobio.2013.06.001 23851106

[B23] Di Cera E. (2008). Thrombin. Mol. Asp. Med. 29, 203–254. 10.1016/j.mam.2008.01.001 PMC2491495 18329094

[B24] Donovan F. M. Cunningham D. D. (1998). Signaling pathways involved in thrombin-induced cell protection. J. Biol. Chem. 273, 12746–12752. 10.1074/jbc.273.21.12746 9582299

[B25] Donovan F. M. Pike C. J. Cotman C. W. Cunningham D. D. (1997). Thrombin induces apoptosis in cultured neurons and astrocytes via a pathway requiring tyrosine kinase and RhoA activities. J. Neurosci. 17, 5316–5326. 10.1523/JNEUROSCI.17-14-05316.1997 9204916 PMC6793831

[B26] Fang H. Chen J. Lin S. Wang P. Wang Y. Xiong X. (2014). CD36-mediated hematoma absorption following intracerebral hemorrhage: negative regulation by TLR4 signaling. J. Immunol. 192, 5984–5992. 10.4049/jimmunol.1400054 24808360 PMC4049082

[B27] Figueroa B. E. Keep R. F. Betz A. L. Hoff J. T. (1998). Plasminogen activators potentiate thrombin-induced brain injury. Stroke 29, 1202–1207. 10.1161/01.str.29.6.1202 9626295

[B28] Fleming I. Bauersachs J. Busse R. (1997). Calcium-dependent and calcium-independent activation of the endothelial NO synthase. J. Vasc. Res. 34, 165–174. 10.1159/000159220 9226298

[B29] Florczak-Rzepka M. Grond-Ginsbach C. Montaner J. Steiner T. (2012). Matrix metalloproteinases in human spontaneous intracerebral hemorrhage: an update. Cerebrovasc. Dis. 34, 249–262. 10.1159/000341686 23052179

[B30] Fujimoto S. Katsuki H. Kume T. Akaike A. (2006). Thrombin-induced delayed injury involves multiple and distinct signaling pathways in the cerebral cortex and the striatum in organotypic slice cultures. Neurobiol. Dis. 22, 130–142. 10.1016/j.nbd.2005.10.008 16330215

[B31] Fujimoto S. Katsuki H. Ohnishi M. Takagi M. Kume T. Akaike A. (2007). Thrombin induces striatal neurotoxicity depending on mitogen-activated protein kinase pathways *in vivo* . Neuroscience 144, 694–701. 10.1016/j.neuroscience.2006.09.049 17084034

[B32] Fujimoto S. Katsuki H. Ohnishi M. Takagi M. Kume T. Akaike A. (2008). Plasminogen potentiates thrombin cytotoxicity and contributes to pathology of intracerebral hemorrhage in rats. J. Cereb. Blood Flow. Metab. 28, 506–515. 10.1038/sj.jcbfm.9600547 17940541

[B33] Fu Q. Cheng J. Gao Y. Zhang Y. Chen X. Xie J. (2015). Protease-activated receptor 4: a critical participator in inflammatory response. Inflammation 38, 886–895. 10.1007/s10753-014-9999-6 25120239

[B34] GarcíA P. S. Ciavatta V. T. Fidler J. A. Woodbury A. Levy J. H. Tyor W. R. (2015). Concentration-dependent dual role of thrombin in protection of cultured rat cortical neurons. Neurochem. Res. 40, 2220–2229. 10.1007/s11064-015-1711-1 26342829 PMC4644093

[B35] Gingrich M. B. Junge C. E. Lyuboslavsky P. Traynelis S. F. (2000). Potentiation of NMDA receptor function by the serine protease thrombin. J. Neurosci. 20, 4582–4595. 10.1523/JNEUROSCI.20-12-04582.2000 10844028 PMC6772448

[B36] Ginhoux F. Greter M. Leboeuf M. Nandi S. See P. Gokhan S. (2010). Fate mapping analysis reveals that adult microglia derive from primitive macrophages. Science 330, 841–845. 10.1126/science.1194637 20966214 PMC3719181

[B37] Golderman V. Ben-Shimon M. Maggio N. Dori A. Gofrit S. G. Berkowitz S. (2022). Factor VII, EPCR, aPC Modulators: novel treatment for neuroinflammation. J. Neuroinflammation 19, 138. 10.1186/s12974-022-02505-y 35690769 PMC9187898

[B38] Gong C. Hoff J. T. Keep R. F. (2000). Acute inflammatory reaction following experimental intracerebral hemorrhage in rat. Brain Res. 871, 57–65. 10.1016/s0006-8993(00)02427-6 10882783

[B39] Gong Y. XI G. H. Keep R. F. Hoff J. T. Hua Y. (2005). Complement inhibition attenuates brain edema and neurological deficits induced by thrombin. Acta Neurochir. Suppl. 95, 389–392. 10.1007/3-211-32318-x_79 16463887

[B40] Gong Y. XI G. Hu H. Gu Y. Huang F. Keep R. F. (2008). Increase in brain thrombin activity after experimental intracerebral hemorrhage. Acta Neurochir. Suppl. 105, 47–50. 10.1007/978-3-211-09469-3_10 19066081

[B41] Guerrero J. SantibañEZ J. F. GonzáLEZ A. MartíNEZ J. (2004). EGF receptor transactivation by urokinase receptor stimulus through a mechanism involving Src and matrix metalloproteinases. Exp. Cell Res. 292, 201–208. 10.1016/j.yexcr.2003.08.011 14720519

[B42] Han X. Nieman M. T. (2020). The domino effect triggered by the tethered ligand of the protease activated receptors. Thromb. Res. 196, 87–98. 10.1016/j.thromres.2020.08.004 32853981 PMC7686079

[B43] Heuberger D. M. Schuepbach R. A. (2019). Protease-activated receptors (PARs): mechanisms of action and potential therapeutic modulators in PAR-driven inflammatory diseases. Thromb. J. 17, 4. 10.1186/s12959-019-0194-8 30976204 PMC6440139

[B44] Hijioka M. Futokoro R. Ohto-Nakanishi T. Nakanishi H. Katsuki H. Kitamura Y. (2020). Microglia-released leukotriene B(4) promotes neutrophil infiltration and microglial activation following intracerebral hemorrhage. Int. Immunopharmacol. 85, 106678. 10.1016/j.intimp.2020.106678 32544870

[B45] Hooijmans C. R. Rovers M. M. De Vries R. B. Leenaars M. Ritskes-Hoitinga M. Langendam M. W. (2014). SYRCLE's risk of bias tool for animal studies. BMC Med. Res. Methodol. 14, 43. 10.1186/1471-2288-14-43 24667063 PMC4230647

[B46] Hostettler I. C. Seiffge D. J. Werring D. J. (2019). Intracerebral hemorrhage: an update on diagnosis and treatment. Expert Rev. Neurother. 19, 679–694. 10.1080/14737175.2019.1623671 31188036

[B47] Hua Y. Keep R. F. Hoff J. T. XI G. (2003). Thrombin preconditioning attenuates brain edema induced by erythrocytes and iron. J. Cereb. Blood Flow. Metab. 23, 1448–1454. 10.1097/01.WCB.0000090621.86921.D5 14663340

[B48] Hua Y. Keep R. F. Hoff J. T. XI G. (2007). Brain injury after intracerebral hemorrhage: the role of thrombin and iron. Stroke 38, 759–762. 10.1161/01.STR.0000247868.97078.10 17261733

[B49] Hua Y. XI G. Keep R. F. Hoff J. T. (2000). Complement activation in the brain after experimental intracerebral hemorrhage. J. Neurosurg. 92, 1016–1022. 10.3171/jns.2000.92.6.1016 10839264

[B50] Hu E. Hu W. Yang A. Zhou H. Zhou J. Luo J. (2019a). Thrombin promotes pericyte coverage by Tie2 activation in a rat model of intracerebral hemorrhage. Brain Res. 1708, 58–68. 10.1016/j.brainres.2018.12.003 30527680

[B51] Hui W. N. Chu R. T. Shao G. P. Huang W. Li X. D. &Hui G. Z. (2013). Relationship between brain cells apoptosis and thrombin in the brain tissue around hematoma. Jiangsu Med. J. 39, 2713–2716+2801. 10.19460/j.cnki.0253-3685.2013.22.020

[B52] Hu S. Wu G. Ding X. Zhang Y. (2016). Thrombin preferentially induces autophagy in glia cells in the rat central nervous system. Neurosci. Lett. 630, 53–58. 10.1016/j.neulet.2016.07.023 27431453

[B53] Hu S. Wu G. Zheng J. Liu X. Zhang Y. (2019b). Astrocytic thrombin-evoked VEGF release is dependent on p44/42 MAPKs and PAR1. Biochem. Biophys. Res. Commun. 509, 585–589. 10.1016/j.bbrc.2018.12.168 30606478

[B54] Hu S. XI G. Jin H. He Y. Keep R. F. Hua Y. (2011). Thrombin-induced autophagy: a potential role in intracerebral hemorrhage. Brain Res. 1424, 60–66. 10.1016/j.brainres.2011.09.062 22015349 PMC3219926

[B55] Hu X. Leak R. K. Shi Y. Suenaga J. Gao Y. Zheng P. (2015). Microglial and macrophage polarization—new prospects for brain repair. Nat. Rev. Neurol. 11, 56–64. 10.1038/nrneurol.2014.207 25385337 PMC4395497

[B56] Kawakita K. Kawai N. Kuroda Y. Yasashita S. Nagao S. (2006). Expression of matrix metalloproteinase-9 in thrombin-induced brain edema formation in rats. J. Stroke Cerebrovasc. Dis. 15, 88–95. 10.1016/j.jstrokecerebrovasdis.2006.01.002 17904058

[B57] Kearns K. N. Ironside N. Park M. S. Worrall B. B. Southerland A. M. Chen C. J. (2021). Neuroprotective therapies for spontaneous intracerebral hemorrhage. Neurocrit Care 35, 862–886. 10.1007/s12028-021-01311-3 34341912

[B58] Krenzlin H. Gresser E. Jussen D. Riede N. Taylor L. Vogelaar C. F. (2020). The cerebral thrombin system is activated after intracerebral hemorrhage and contributes to secondary lesion growth and poor neurological outcome in C57Bl/6 mice. J. Neurotrauma 37, 1481–1490. 10.1089/neu.2019.6582 31830857

[B59] Kuschel A. Simon P. Tug S. (2012). Functional regulation of HIF-1α under normoxia--is there more than post-translational regulation? J. Cell Physiol. 227, 514–524. 10.1002/jcp.22798 21503885

[B60] Lan X. Han X. Li Q. Yang Q. W. Wang J. (2017). Modulators of microglial activation and polarization after intracerebral haemorrhage. Nat. Rev. Neurol. 13, 420–433. 10.1038/nrneurol.2017.69 28524175 PMC5575938

[B61] Lee K. R. Kawai N. Kim S. Sagher O. Hoff J. T. (1997). Mechanisms of edema formation after intracerebral hemorrhage: effects of thrombin on cerebral blood flow, blood-brain barrier permeability, and cell survival in a rat model. J. Neurosurg. 86, 272–278. 10.3171/jns.1997.86.2.0272 9010429

[B62] Liesz A. Middelhoff M. Zhou W. Karcher S. Illanes S. Veltkamp R. (2011). Comparison of humoral neuroinflammation and adhesion molecule expression in two models of experimental intracerebral hemorrhage. Exp. Transl. Stroke Med. 3, 11. 10.1186/2040-7378-3-11 21967730 PMC3195108

[B63] Liu D. Z. Ander B. P. Xu H. Shen Y. Kaur P. Deng W. (2010). Blood-brain barrier breakdown and repair by Src after thrombin-induced injury. Ann. Neurol. 67, 526–533. 10.1002/ana.21924 20437588 PMC2919346

[B64] Li X. Zhu Z. Gao S. Zhang L. Cheng X. Li S. (2019). Inhibition of fibrin formation reduces neuroinflammation and improves long-term outcome after intracerebral hemorrhage. Int. Immunopharmacol. 72, 473–478. 10.1016/j.intimp.2019.04.029 31039464

[B65] Machida T. Takata F. Matsumoto J. Takenoshita H. Kimura I. Yamauchi A. (2015). Brain pericytes are the most thrombin-sensitive matrix metalloproteinase-9-releasing cell type constituting the blood-brain barrier *in vitro* . Neurosci. Lett. 599, 109–114. 10.1016/j.neulet.2015.05.028 26002077

[B66] Mckeage K. Plosker G. L. (2001). Argatroban. Drugs 61, 515–524. 10.2165/00003495-200161040-00005 11324681

[B67] Menzies S. A. Betz A. L. Hoff J. T. (1993). Contributions of ions and albumin to the formation and resolution of ischemic brain edema. J. Neurosurg. 78, 257–266. 10.3171/jns.1993.78.2.0257 8421208

[B68] Mu Q. Wang L. Hang H. Liu C. Wu G. (2017). Rosiglitazone pretreatment influences thrombin-induced phagocytosis by rat microglia via activating PPARγ and CD36. Neurosci. Lett. 651, 159–164. 10.1016/j.neulet.2017.04.038 28445772

[B69] Nagatsuna T. Nomura S. Suehiro E. Fujisawa H. Koizumi H. Suzuki M. (2005). Systemic administration of argatroban reduces secondary brain damage in a rat model of intracerebral hemorrhage: histopathological assessment. Cerebrovasc. Dis. 19, 192–200. 10.1159/000083466 15665510

[B70] Nakamura T. XI G. Park J. W. Hua Y. Hoff J. T. Keep R. F. (2005). Holo-transferrin and thrombin can interact to cause brain damage. Stroke 36, 348–352. 10.1161/01.STR.0000153044.60858.1b 15637325

[B71] Niu M. Dai X. Zou W. Yu X. Teng W. Chen Q. (2017). Autophagy, endoplasmic reticulum stress and the unfolded protein response in intracerebral hemorrhage. Transl. Neurosci. 8, 37–48. 10.1515/tnsci-2017-0008 28729917 PMC5444040

[B72] Noda D. Kurauchi Y. Hisatsune A. Seki T. Katsuki H. (2020). Interactions between rat cortico-striatal slice cultures and neutrophil-like HL60 cells under thrombin challenge: toward elucidation of pathological events in intracerebral hemorrhage. J. Pharmacol. Sci. 142, 116–123. 10.1016/j.jphs.2019.12.006 31924407

[B73] Ohnishi M. Katsuki H. Fujimoto S. Takagi M. Kume T. Akaike A. (2007). Involvement of thrombin and mitogen-activated protein kinase pathways in hemorrhagic brain injury. Exp. Neurol. 206, 43–52. 10.1016/j.expneurol.2007.03.030 17498698

[B74] Ossovskaya V. S. Bunnett N. W. (2004). Protease-activated receptors: contribution to physiology and disease. Physiol. Rev. 84, 579–621. 10.1152/physrev.00028.2003 15044683

[B75] Paul J. Strickland S. Melchor J. P. (2007). Fibrin deposition accelerates neurovascular damage and neuroinflammation in mouse models of Alzheimer's disease. J. Exp. Med. 204, 1999–2008. 10.1084/jem.20070304 17664291 PMC2118680

[B76] Persidsky Y. Ramirez S. H. Haorah J. Kanmogne G. D. (2006). Blood-brain barrier: structural components and function under physiologic and pathologic conditions. J. Neuroimmune Pharmacol. 1, 223–236. 10.1007/s11481-006-9025-3 18040800

[B77] Ren H. Han R. Chen X. Liu X. Wan J. Wang L. (2020). Potential therapeutic targets for intracerebral hemorrhage-associated inflammation: an update. J. Cereb. Blood Flow. Metab. 40, 1752–1768. 10.1177/0271678X20923551 32423330 PMC7446569

[B78] Ryu J. K. Petersen M. A. Murray S. G. Baeten K. M. Meyer-Franke A. Chan J. P. (2015). Blood coagulation protein fibrinogen promotes autoimmunity and demyelination via chemokine release and antigen presentation. Nat. Commun. 6, 8164. 10.1038/ncomms9164 26353940 PMC4579523

[B79] Shao Z. Tu S. Shao A. (2019). Pathophysiological mechanisms and potential therapeutic targets in intracerebral hemorrhage. Front. Pharmacol. 10, 1079. 10.3389/fphar.2019.01079 31607923 PMC6761372

[B80] Striggow F. Riek M. Breder J. Henrich-Noack P. Reymann K. G. Reiser G. (2000). The protease thrombin is an endogenous mediator of hippocampal neuroprotection against ischemia at low concentrations but causes degeneration at high concentrations. Proc. Natl. Acad. Sci. U. S. A. 97, 2264–2269. 10.1073/pnas.040552897 10681455 PMC15789

[B81] Sun Z. Zhao Z. Zhao S. Sheng Y. Zhao Z. Gao C. (2009). Recombinant hirudin treatment modulates aquaporin-4 and aquaporin-9 expression after intracerebral hemorrhage *in vivo* . Mol. Biol. Rep. 36, 1119–1127. 10.1007/s11033-008-9287-3 18574711

[B82] Suresh S. Begum R. F. Singh S. A. (2022). Anthocyanin as a therapeutic in Alzheimer's disease: a systematic review of preclinical evidences. Ageing Res. Rev. 76, 101595. 10.1016/j.arr.2022.101595 35217244

[B83] Tampo Y. Kotamraju S. Chitambar C. R. Kalivendi S. V. Keszler A. Joseph J. (2003). Oxidative stress-induced iron signaling is responsible for peroxide-dependent oxidation of dichlorodihydrofluorescein in endothelial cells: role of transferrin receptor-dependent iron uptake in apoptosis. Circ. Res. 92, 56–63. 10.1161/01.res.0000048195.15637.ac 12522121

[B84] Templeton D. M. Liu Y. (2003). Genetic regulation of cell function in response to iron overload or chelation. Biochim. Biophys. Acta 1619, 113–124. 10.1016/s0304-4165(02)00497-x 12527106

[B85] Tschoe C. Bushnell C. D. Duncan P. W. Alexander-Miller M. A. Wolfe S. Q. (2020). Neuroinflammation after intracerebral hemorrhage and potential therapeutic targets. J. Stroke 22, 29–46. 10.5853/jos.2019.02236 32027790 PMC7005353

[B86] Wang C. W. Klionsky D. J. (2003). The molecular mechanism of autophagy. Mol. Med. 9, 65–76. 10.1007/bf03402040 12865942 PMC1430730

[B87] Wang H. Ricklin D. Lambris J. D. (2017). Complement-activation fragment C4a mediates effector functions by binding as untethered agonist to protease-activated receptors 1 and 4. Proc. Natl. Acad. Sci. U. S. A. 114, 10948–10953. 10.1073/pnas.1707364114 28973891 PMC5642699

[B88] Wang X. Bleher R. Brown M. E. Garcia J. G. Dudek S. M. Shekhawat G. S. (2015). Nano-biomechanical study of spatio-temporal cytoskeleton rearrangements that determine subcellular mechanical properties and endothelial permeability. Sci. Rep. 5, 11097. 10.1038/srep11097 26086333 PMC4650616

[B89] Wang Z. Wang Y. K. Feng W. Zhang J. W. (2016). Research progress on the blood-brain barrier dysfunction after intracerebral hemorrhage. J. Mol. Imagin 39, 420–424. 10.3969/j.issn.1674-4500.2016.04.23

[B90] Wang Z. Zhou F. Dou Y. Tian X. Liu C. Li H. (2018). Melatonin alleviates intracerebral hemorrhage-induced secondary brain injury in rats via suppressing apoptosis, inflammation, oxidative stress, DNA damage, and mitochondria injury. Transl. Stroke Res. 9, 74–91. 10.1007/s12975-017-0559-x 28766251 PMC5750335

[B91] Wan S. Cheng Y. Jin H. Guo D. Hua Y. Keep R. F. (2016). Microglia activation and polarization after intracerebral hemorrhage in mice: the role of protease-activated receptor-1. Transl. Stroke Res. 7, 478–487. 10.1007/s12975-016-0472-8 27206851 PMC5065741

[B92] Wilkinson D. A. Pandey A. S. Thompson B. G. Keep R. F. Hua Y. XI G. (2018). Injury mechanisms in acute intracerebral hemorrhage. Neuropharmacology 134, 240–248. 10.1016/j.neuropharm.2017.09.033 28947377 PMC6027647

[B93] Wu C. H. Yang R. L. Li H. Z. Wu S. Y. Huang S. Y. Lei H. X. (2006). “Significance of changes in plasma and hematoma fluid TAT levels in patients with intracerebral hemorrhage,” in Fifth organizational meeting of the Chinese journal of emergency medicine (Changchun, Jilin, China: Spinger).

[B94] Wu L. X. Zhan Q. Q. Liu P. Zheng H. Q. Liu M. X. Min J. (2022). LncRNA TCONS_00145741 knockdown prevents thrombin-induced M1 differentiation of microglia in intracerebral hemorrhage by enhancing the interaction between DUSP6 and JNK. Front. Cell Dev. Biol. 9, 684842. 10.3389/fcell.2021.684842 35127692 PMC8809462

[B95] Wu C. Yan X. Liao Y. Liao L. Huang S. Zuo Q. (2019). Increased perihematomal neuron autophagy and plasma thrombin-antithrombin levels in patients with intracerebral hemorrhage: an observational study. Med. Baltim. 98, e17130. 10.1097/MD.0000000000017130 PMC6775380 31574813

[B96] Wu H. Wu T. Xu X. Wang J. Wang J. (2011). Iron toxicity in mice with collagenase-induced intracerebral hemorrhage. J. Cereb. Blood Flow. Metab. 31, 1243–1250. 10.1038/jcbfm.2010.209 21102602 PMC3099628

[B97] Wu H. Zhang Z. Li Y. Zhao R. Li H. Song Y. (2010). Time course of upregulation of inflammatory mediators in the hemorrhagic brain in rats: correlation with brain edema. Neurochem. Int. 57, 248–253. 10.1016/j.neuint.2010.06.002 20541575 PMC2910823

[B98] Xue M. Del Bigio M. R. (2001). Acute tissue damage after injections of thrombin and plasmin into rat striatum. Stroke 32, 2164–2169. 10.1161/hs0901.095408 11546912

[B99] Xue M. Hollenberg M. D. Yong V. W. (2006). Combination of thrombin and matrix metalloproteinase-9 exacerbates neurotoxicity in cell culture and intracerebral hemorrhage in mice. J. Neurosci. 26, 10281–10291. 10.1523/JNEUROSCI.2806-06.2006 17021183 PMC6674619

[B100] Ye F. Yang J. Hua Y. Keep R. F. XI G. (2023). Novel approach to visualize microglia death and proliferation after intracerebral hemorrhage in mice. Stroke 53, e472–e476. 10.1161/STROKEAHA.122.040302 PMC9613600 36148656

[B101] Zeng X. Zhang Y. Kwong J. S. Zhang C. Li S. Sun F. (2015). The methodological quality assessment tools for preclinical and clinical studies, systematic review and meta-analysis, and clinical practice guideline: a systematic review. J. Evid. Based Med. 8, 2–10. 10.1111/jebm.12141 25594108

[B102] Zhang Z. Y. Qi J. P. Zhu H. Song Y. J. Wu H. Jia Y. (2010). Expression of thrombin and its associated protein in cerebellum of human and rat after intracerebral hemorrhage. Chin. Med. J. Engl. 123, 2077–2081. 10.3760/cma.j.issn.0366-6999.2010.15.022 20819545

[B103] Zhang Y. Khan S. Liu Y. Wu G. Yong V. W. Xue M. (2022). Oxidative stress following intracerebral hemorrhage: from molecular mechanisms to therapeutic targets. Front. Immunol. 13, 847246. 10.3389/fimmu.2022.847246 35355999 PMC8959663

[B104] Zhao P. Metcalf M. Bunnett N. W. (2014). Biased signaling of protease-activated receptors. Front. Endocrinol. (Lausanne) 5, 67. 10.3389/fendo.2014.00067 24860547 PMC4026716

[B105] Zheng H. Chen C. Zhang J. Hu Z. (2016). Mechanism and therapy of brain edema after intracerebral hemorrhage. Cerebrovasc. Dis. 42, 155–169. 10.1159/000445170 27110940

[B106] Zhou H. J. Tang T. Cui H. J. Yang A. L. Luo J. K. Lin Y. (2012). Thrombin-triggered angiogenesis in rat brains following experimental intracerebral hemorrhage. J. Neurosurg. 117, 920–928. 10.3171/2012.8.JNS112152 22957530

[B107] Zhou Z. H. Li W. (2002). Thrombin and neuro-cell apoptosis in intracerebral hemorrhage. Graduate Med. J., 160–162. 10.16571/j.cnki.1008-8199.2002.02.025

[B108] Zhou Z. H. Qu F. Zhang C. D. (2011). Systemic administration of argatroban inhibits protease-activated receptor-1 expression in perihematomal tissue in rats with intracerebral hemorrhage. Brain Res. Bull. 86, 235–238. 10.1016/j.brainresbull.2011.07.012 21803126

[B109] Zhu H. Wang Z. Yu J. Yang X. He F. Liu Z. (2019). Role and mechanisms of cytokines in the secondary brain injury after intracerebral hemorrhage. Prog. Neurobiol. 178, 101610. 10.1016/j.pneurobio.2019.03.003 30923023

